# Circulating Tumor DNA is Unreliable to Detect Somatic Gene Alterations in Gastrointestinal Peritoneal Carcinomatosis

**DOI:** 10.1245/s10434-022-12399-y

**Published:** 2022-08-18

**Authors:** Brittany G. Sullivan, Angelina Lo, Jingjing Yu, Amber Gonda, Farideh Dehkordi-Vakil, Farshid Dayyani, Maheswari Senthil

**Affiliations:** 1 Division of Surgical Oncology, Department of Surgery, University of California Irvine, Orange, CA USA; 2 Division of Hematology-Medical Oncology, Department of Medicine, University of California Irvine, Orange, CA USA

## Abstract

**Introduction:**

Tumor agnostic circulating tumor DNA (ctDNA) is routinely used to guide treatment decisions in gastrointestinal (GI) cancers, especially metastatic cancers. The amount of ctDNA detected in plasma is affected by stage, tumor burden, and tumor vascularization. We hypothesized that peritoneal carcinomatosis (PC) is associated with lower ctDNA levels than other metastatic sites in GI cancers due to the plasma–peritoneal barrier.

**Methods:**

We conducted a retrospective analysis of patients with stage II–IV GI cancers treated at our institution between 2015 and 2020 with available panel-based ctDNA results (Guardant 360^TM^). ctDNA analysis was performed on early and pretreatment samples. We compared the reported maximum variant allele frequency (mVAF) of somatic mutations across metastatic sites.

**Results:**

Of the 279 patients with GI cancers (colorectal, upper GI, pancreaticobiliary), 212 had stage IV disease (PC: *n* = 61; visceral metastases: *n* = 138; other metastases: *n* = 13). Mean mVAF increased with increasing stages of disease (stage II: 3.6 ± 7; stage III: 6.4 ± 10; stage IV: 28.0 ± 51; *p* < 0.01). Among patients with stage IV disease, PC was associated with lower ctDNA levels independent of primary tumor site (PC only: 12.1%; PC+ visceral metastases: 26.8%; and visceral metastases only: 35.0%; *p* < 0.01). In a subset of patients (*n* = 27, matched pair analysis of genomic alterations (GAs) showed fewer GAs were detected in plasma compared with tissue.

**Conclusions:**

PC of GI origin is associated with significantly lower ctDNA levels compared with visceral metastasis. Caution is warranted when interpreting ctDNA results from patients with PC due to lower sensitivity for detecting actionable mutations.

In the United States, digestive system cancers are estimated to account for over 343,000 new cancer cases and 171,000 deaths in 2022, making it the most common cause of cancer mortality.^1^ Since cancer-related deaths are largely due to metastatic disease, improvement in the prediction, detection, and treatment of metastatic disease is crucial to improve outcomes. Over the last decade, liquid biopsies that detect circulating tumor DNA (ctDNA), which are short nucleic acid fragments shed from tumor cells into the circulation,^2^ have improved our ability to predict recurrence, detect disease, and assess treatment response in gastrointestinal (GI) cancers, among other cancers.^3^ Tumor-agnostic ctDNA assays seek to identify known genomic alterations (GAs) that have proven predictive or prognostic value with high specificity.^4^ In 2014, Bettegowda et al. reported that ctDNA was detected in >75% of advanced cancers and identified clinically relevant KRAS mutation in metastatic colorectal cancer with a 99.2% specificity.^5^ Subsequent studies have established that ctDNA has a high positive predictive value for disease recurrence in stage II–III colon cancer due to its high sensitivity to detect molecular residual disease (MRD) post resection.^6^ In the current era of personalized medicine with precision therapies, detecting GAs and MRD through a minimally invasive approach using ctDNA liquid biopsy has become an integral part of cancer care. Moreover, the reported utility of ctDNA to identify recurrence and monitor treatment response through serial measurements combined with the benefit of avoiding invasive tissue biopsies are the underlying reasons as to why there are over 350 clinical trials, with ctDNA either as a marker or endpoint for cancer treatment, registered in the Clinical Trials Network (ClinicalTrials.gov) at the time of this writing.

Despite the promising potential of ctDNA liquid biopsy, there are important limitations. The amount of ctDNA detected is influenced by disease burden, location of disease, treatment of disease, and tumor vascularization.^2,7–10^ Peritoneal carcinomatosis (PC), an aggressive form of metastatic spread in GI cancers, with diffuse involvement of the peritoneal lining, represents a distinct form of metastatic disease as these tumors tend to be poorly vascularized,^11^ and have less communication with systemic circulation due to the peritoneal-plasma barrier.^12–14^ Despite the biologic differences of PC from other metastases, most ctDNA studies combine the metastatic sites. Recently, there have been conflicting reports in the literature about the utility of ctDNA in the setting of PC.^15–17^ Due to the proclivity of GI cancers to metastasize to the peritoneum, and the rapidly evolving ctDNA-informed treatment approaches in GI cancers, there is an urgent and critical need to clarify the role of ctDNA in GI PC. We sought to evaluate the quantity of plasma ctDNA and the utility of ctDNA to detect tumor-specific GAs in PC metastasis compared with non-PC metastasis in patients with a wide variety of GI cancers.

## Methods

This retrospective, single-center study included patients who were aged 18 years and older and diagnosed with stage II–IV GI malignancy from 2015 to 2020 with available panel-based ctDNA results (Guardant 360^TM^). GI malignancies were defined as any malignancy of the esophagus, stomach, small bowel, colon, rectum, anus, liver, bile ducts, gallbladder, or pancreas. The Guardant 360^TM^ platform is a US FDA-approved, 73-gene next-generation sequencing (NGS) test that detects plasma ctDNA fragments along with germline and other somatic cell-free DNA (cfDNA).^18^ A limitation of the Guardant 360^TM^ platform is its decreased sensitivity to detect ctDNA in mucinous tumors.^19^ ctDNA analysis was performed on blood samples collected either pretreatment (chemotherapy, radiation, or surgery) or early treatment (within 1 month of the initiation of treatment). Majority of patients with early treatment blood draws had stage II–III disease. All patients with stage IV disease had evidence of disease at the time of ctDNA blood collection.

Overall, 279 patients who met the inclusion criteria were identified. Patient demographics, tumor characteristics, ctDNA collection date and results, and treatment information were collected by chart review. In patients with PC, the number of intra-abdominal regions involved (0–8) were recorded by radiological assessment of cross-sectional imaging (computed tomography/magnetic resonance imaging [CT/MRI]) performed around the time of the ctDNA blood draw as previously described.^20^

The primary origin of cancers was divided into three groups: upper GI, which included esophageal, gastric, and small bowel; colorectal, which included colon, rectum, and anus; and hepato-pancreato-biliary (HPB), which included pancreas, primary liver, bile duct, and gallbladder. Neuroendocrine tumors, lymphoma, and GI stromal tumors were excluded. Metastatic sites were divided into four groups: visceral (liver and/or lung), PC only, PC + visceral, and other (bone and/or brain). One patient was classified as PC + other.

Plasma ctDNA NGS data obtained from Guardant 360^TM^ included maximum variant allele frequency (mVAF) of somatic alterations, gene amplifications, microsatellite instability status, and tumor mutational burden. The ratio of tumor DNA to cfDNA is reported as variant allele frequency (VAF) percentage. The mVAF reflects the amount of ctDNA present within a sample and can be used to quantify the amount of ctDNA shed by tumor into the circulation.^9^ The Kruskal–Wallis test was used to compare the reported mVAF of somatic mutations of the three groups, across all metastatic sites and for stage of disease. In a subset of patients with PC who had both plasma ctDNA NGS and tissue NGS analysis, comparison of GAs between plasma and tissue was performed to assess concordance.

This study was conducted in accordance with the Declaration of Helsinki (as revised in 2013). The current study was approved by the Institutional Review Board at the University of California Irvine (protocol #2020-6196) and Saint Luke’s Health System (protocol #001CTDNA21). Individual consent for this retrospective anonymized study was waived.

## Results

### Patient and Tumor Variables

Patient demographics and tumor characteristics are described in Table 1. A total of 279 patients with stage II–IV GI cancer who had plasma ctDNA analysis were included in the study; median age was 61 years (range 26–98), 57.0% of patients were male, and the majority of patients self-identified their race as non-Hispanic White (*n* = 106, 38%). The most frequent primary tumor site was colorectal (*n* = 115, 41.2%) followed by HPB (31.5%) and upper GI (27.2%). The majority of patients had stage IV disease (*n* = 212/279; 76%) and the most common site of metastasis was to the visceral organs (*n* = 138, 49.5%). Sixty-one (21.9%) patients had PC, and of those with PC, the majority had a primary tumor site of upper GI (*n* = 31, 50.8%), followed by colorectal (*n* = 19, 31.1%), and HPB (*n* = 11, 18.0%).

**Table 1:** Patient demographics and tumor characteristics [*n* = 279]

Median age, years (range)	61 (26–98)
*Race and ethnicity n(%)*	
Asian/Pacific Islander	79 (28.3)
Non-Hispanic Black	6 (2.15)
Hispanic	55 (19.7)
Non-Hispanic White	106 (38.0)
Other	33 (11.8)
*Sex*	
Female	120 (43.0)
Male	159 (57.0)
*AJCC stage*	
II	19 (6.81)
III	48 (17.2)
IV	212 (76.0)
*PC*	
Yes	61 (21.9)
No	218 (78.1)
*Type of cancer*	
Colorectal	115 (41.2)
HPB	88 (31.5)
Upper GI	76 (27.2)
*Site of metastasis*	
Visceral	138 (49.5)
PC only	50 (17.9)
PC + visceral	10 (3.60)
PC + other	1 (0.36)
Other	13 (4.66)
*Primary tumor present (yes)*	
Stage II	11 (3.94)
Stage III	36 (12.9)
Stage IV	143 (51.3)
Visceral	96 (67.1)
PC only	29 (20.3)
PC + visceral	7 (4.90)
PC + other	1 (0.70)
Other	10 (7.0)

Data are expressed as *n* (%) unless otherwise specified

*AJCC* American Joint Committee on Cancer, *PC* peritoneal carcinomatosis, *HPB* hepato-pancreato-biliary, *GI* gastrointestinal

### Circulating Tumor DNA (ctDNA) Levels Based on Stage and Primary Tumor Site

**Fig. 1:**
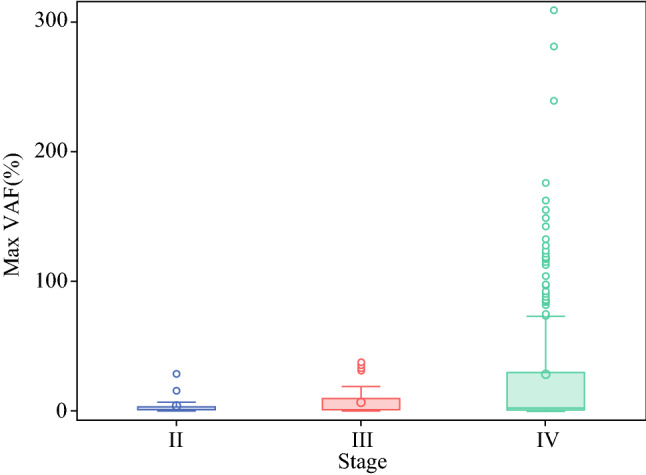
mVAF stratified by stage of disease. ctDNA levels were stratified by stage of disease. The mean mVAF increased with increasing stage of disease: stage II: 3.6 ± 7; stage III: 6.4 ± 10; and stage IV: 28 ± 51.1 (*p* < 0.01). *mVAF* maximum variant allele frequency, *ctDNA* circulating tumor DNA

**Fig. 2:**
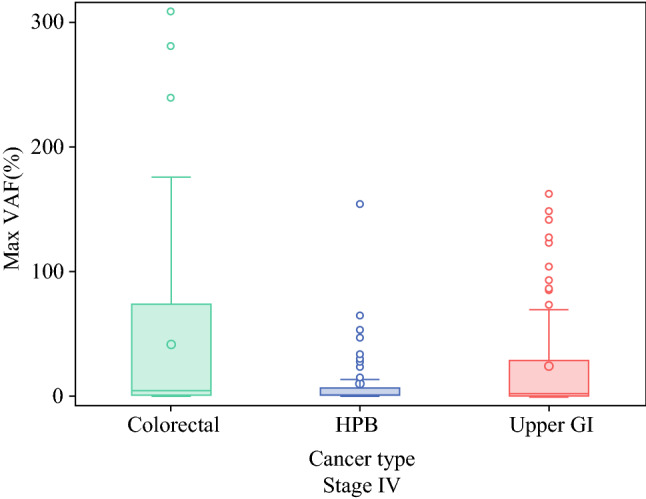
mVAF comparison by primary tumor site in stage IV disease. ctDNA levels were compared in patients with stage IV disease (*n* = 212) based on primary tumor site. Stage IV colorectal cancer had the highest level of ctDNA, followed by upper GI and HPB (41.1 ± 63.8, 24.1 ± 41.6, and 9.91 ± 23.9, respectively; *p* < 0.05). *mVAF* maximum variant allele frequency, *ctDNA* circulating tumor DNA, *GI* gastrointestinal, *HPB* hepato-pancreato-biliary

We compared the amount of plasma ctDNA, expressed as mean mVAF, across different stages for the entire cohort and based on primary site of tumor origin. Consistent with previous observations, the mean mVAF increased with increasing stages of disease; stage II: 3.6 ± 7; stage III: 6.4 ± 10; and stage IV: 28 ± 51.1 (*p* < 0.01) (Fig. 1). The mean mVAF for all stages of colorectal, HPB, and upper GI primary tumor sites was 34.7 ± 60, 9 ± 19.9, and 21.3 ± 39.2, respectively (*p* = 0.2). On subanalysis, the mean mVAF for stage IV colorectal, HPB, and upper GI was compared. Stage IV colorectal cancer had the highest level of ctDNA, followed by upper GI, and HPB (41.1 ± 63.8, 24.1 ± 41.6, and 9.91 ± 23.9, respectively; *p* < 0.05) (Fig. 2).

### ctDNA Levels in Peritoneal Carcinomatosis (PC) Metastasis Compared with Non-PC Metastasis

All patients with PC had evidence of disease at the time of ctDNA blood draw, with an average of 6 (range 1–8) intra-abdominal regions involved.

**Fig. 3:**
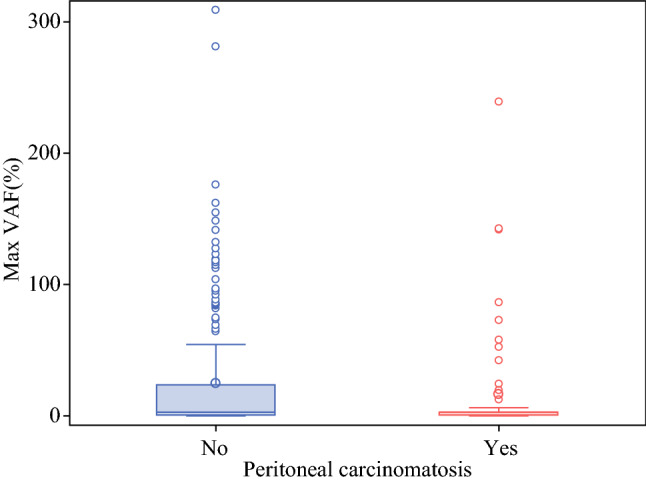
mVAF comparison by PC versus no PC. ctDNA detection was stratified by PC versus no PC for the entire cohort (*n* = 279). Patients with PC had a lower mean mVAF level (16.3 ± 42.2) compared with patients without PC (24.6 ± 47; *p* < 0.01). *mVAF* maximum variant allele frequency, *PC* peritoneal carcinomatosis, *ctDNA* circulating tumor DNA

**Fig. 4:**
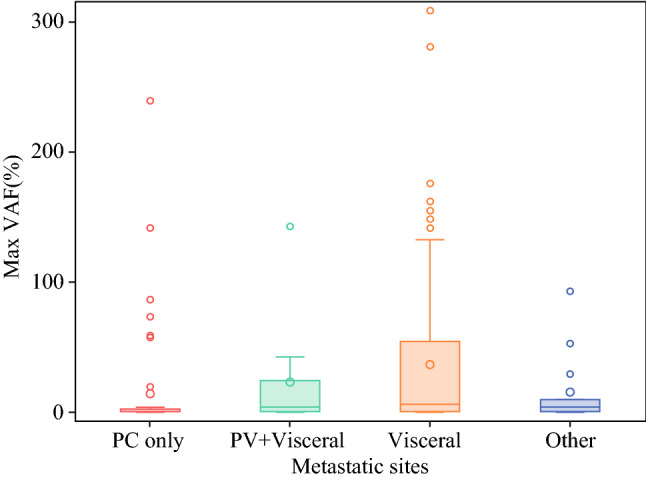
mVAF stratified by metastatic site. ctDNA detection was stratified by metastatic site in patients with stage 4 GI cancers. The mean mVAF was significantly lower in the PC-only group compared with patients with visceral metastases (14.2 ± 42 vs. 36.7 ± 56.5; *p* < 0.01). Patients with PC + visceral metastases had a slightly higher mean mVAF (23.2 ± 44.1) than the PC-only group, but was still significantly lower than the visceral metastases group (*p* < 0.01). *mVAF* maximum variant allele frequency, *ctDNA* circulating tumor DNA, *GI* gastrointestinal, *PC* peritoneal carcinomatosis

We analyzed and compared the quantity of ctDNA levels in patients with (*n* = 61) and without (*n* = 218) PC. Patients with PC had a lower mean mVAF level (16.3 ± 42.2) compared with patients without PC (24.6 ± 47; *p* < 0.01) (Fig. 3); 17/61 (27.9%) patients had no detectable somatic gene mutations in plasma ctDNA NGS. Next, we grouped patients with stage IV disease into four categories based on metastatic site: PC only, PC + visceral, visceral, and other (bone and/or brain), and performed a quantitative comparison (Table 1). The mean mVAF was approximately 2.5 times lower in patients with PC compared with patients with visceral metastases (PC: 14.2 ± 42 vs. visceral: 36.7 ± 56.5; *p* < 0.01) Patients with PC + visceral metastases had a slightly higher mean mVAF of 23.2 ± 44.1, than the PC-only group, but it was still significantly lower than the visceral metastases group (Fig. 4).

### Analysis of Plasma ctDNA Next-Generation Sequencing (NGS) versus Tissue NGS in Patients with PC

A subset analysis was performed on 27 patients with PC who had both ctDNA plasma NGS and tissue NGS performed for the detection of GAs. Overall, ctDNA plasma NGS detected fewer GAs compared with tissue NGS. Of the 27 patients, 15 patients (56%) had no matching GAs between plasma and tissue NGS, and an additional 7 patients (26%) had no pathogenic mutations detected in plasma ctDNA NGS despite being detected on tissue NGS. Combined, these results show an 82% discordance rate in GA detection between plasma ctDNA NGS and tissue NGS in patients with PC.

## Discussion

Our study has two key observations. First, the amount of plasma ctDNA is significantly lower in GI PC compared with visceral metastasis; and second, plasma ctDNA NGS is unreliable to detect GAs in PC, with a very low concordance rate of 18%.

Previous studies have shown that the amount of ctDNA detected in plasma correlates with tumor burden and has prognostic significance. Consistent with the prior observations, we also noted that the ctDNA levels increased with increasing stages, with significantly higher levels of ctDNA detected in stage IV disease. However, among patients with stage IV disease, the group with isolated peritoneal metastasis had the lowest levels of ctDNA despite a large burden of disease seen on CT imaging. Our findings in a wide variety of GI cancers are concordant with the results reported in a study of gastroesophageal adenocarcinoma (*n* = 1627) using 73-gene panel-based ctDNA NGS testing (Guardant 360^TM^). In that study, the authors observed that mVAF was a surrogate marker for disease burden and correlated with number of involved disease sites. However, patients with ‘PC only’ disease had the lowest mean mVAF, with undetectable ctDNA in many patients.^9^ Unlike visceral metastasis, there was no direct correlation between tumor burden and amount of ctDNA in PC. Another study of ctDNA NGS testing prior to cytoreduction surgery in patients with PC reported detection of ctDNA in only 38.8% patients.^14^ There are several plausible reasons for this finding. Peritoneal metastases have chaotic angioarchitecture, and lower vascular density and perfusion compared with the unaffected peritoneum, as shown in the study by Kastelein et al.^21^ Like the blood–brain barrier, the peritoneum has a plasma–peritoneal barrier that separates the peritoneal cavity from the systemic circulation.^12,22^ Furthermore, PC is often associated with mucinous features and the mucin could act as a physical barrier between ctDNA and systemic circulation. Bettegowda et al. reported that neoplasms with mucinous features are associated with low or undetectable ctDNA levels and false-negative gene alterations.^5^ The poor vascularization of these tumors, limited access to the systemic circulation, and physical blockade due to mucinous features are proposed mechanisms as to why patients with PC have lower plasma ctDNA levels despite a high tumor burden.

The subanalysis of patients with PC who had both plasma ctDNA and tissue NGS analysis showed a low concordance (18%) of matching detectable somatic alterations. In fact, 26% of the patients who had actionable GAs detected on tissue NGS had no pathogenic mutations detected on plasma ctDNA NGS. This is in stark contrast to previous studies in metastatic cancer that have reported a concordance rate as high 80–90% between plasma ctDNA NGS and tumor NGS for GA detection.^19,23^ A study of plasma ctDNA analysis of RAS mutations in metastatic colorectal cancer (*n* = 115) showed significantly low detection of RAS mutant allele fraction in patients with mucinous tumors and isolated peritoneal metastasis (0.1%) compared with other metastasis (4.0%).^24^ In their study of PC, Baumgartner et al. reported a 35.3% concordance rate between tissue and plasma GAs.^14^ The findings of our current study, combined with prior observation, makes it abundantly clear that panel-based ctDNA testing is unreliable to detect somatic gene alterations in PC. Hence, when feasible, tumor NGS should be used in PC to identify GAs. Recently, tumor-informed ctDNA assay (Signatera^TM^) has been favored to detect MRD and assess treatment response due to its higher sensitivity to detect tumor-specific gene mutations.^10^ A recent report published by our group on tumor-informed ctDNA assays in GI cancers showed that ctDNA was detected in only 53% of patients with PC.^10^

The findings of our study have major significance, in light of several clinical trials in GI cancers that are using ctDNA assay to guide treatment decisions. CIRCULATE-US (NCT 05174169) is a randomized clinical trial in which patients with stage III colon cancer who are ctDNA-negative by tumor-informed assay (Signatera^TM^) will be randomized to serial ctDNA monitoring and no adjuvant treatment versus standard systemic therapy. The PEGASUS trial (NCT 04259944) is a single-arm trial in which ctDNA (LUNAR 1 assay, Guardant Health, Redwood City, CA, USA) results will be used to de-escalate treatment in patients with stage III or high-risk stage II colon cancer. It is important to note that in a study of stage III colon cancer that utilized a panel-based ctDNA test to assess the efficacy of adjuvant chemotherapy, only 42% (10/24) of patients with recurrence had postsurgical positive ctDNA.^25^ Given the abundance of evidence that ctDNA is often not detected or is detected in extremely low quantities in patients with PC, significant caution is necessary while enrolling patients with mucinous tumors or tumors with high risk for PC in these studies.

Although ctDNA as a liquid biopsy has limitations in PC, the concept of liquid biopsy to detect and predict treatment response are appropriately suited for the management of PC, as imaging studies and available tumor biomarkers lack sensitivity to detect early PC. Alternate liquid biopsy tests in PC, utilizing peritoneal fluid-based ctDNA^15^ or exosome nanovesicle gene signatures,^26,27^ warrant further exploration. Preliminary work undertaken by our group has led to the discovery of specific exosomal gene expression profiles in colon cancer that may be exploited to develop a liquid biopsy.

The limitations to this study include those inherent to a retrospective database study. Given the retrospective nature of this study, there were variabilities in the timing of ctDNA blood draw, with baseline ctDNA blood drawn in a fraction of patients after initiation of systemic treatment. However, the majority of these patients had stage II–III disease. Hence, the overall result of this study is unlikely to be influenced by this deviation. Furthermore, this was a single-center study and is therefore susceptible to referral bias and population distribution unique to the geographic location of the institution. Finally, this study was not powered to look at the differences between the PC and no PC groups based on primary tumor site. However, primary tumor site-specific studies have confirmed that in gastric and colorectal cancer, PC has lower ctDNA levels compared with other metastatic sites.^9,28^ Despite these limitations, this was the largest study to date of plasma ctDNA analysis in patients with PC with a diverse race and ethnic representation and wide range of GI cancers, and might better reflect GI cancers *in toto* compared with datasets with a predominantly Caucasian population.

## Conclusion

In patients with GI PC, plasma ctDNA NGS is unreliable to detect tumor somatic gene alterations. Furthermore, ctDNA levels are significantly low in PC compared with visceral metastasis, and lack correlation with tumor burden. Hence, caution is warranted in utilizing ctDNA as a therapeutic decision-making tool in patients with PC or who are at high risk for PC.
